# Th17 responses and natural IgM antibodies are related to gut microbiota composition in systemic lupus erythematosus patients

**DOI:** 10.1038/srep24072

**Published:** 2016-04-05

**Authors:** Patricia López, Banesa de Paz, Javier Rodríguez-Carrio, Arancha Hevia, Borja Sánchez, Abelardo Margolles, Ana Suárez

**Affiliations:** 1 Department of Functional Biology, Immunology Area, Faculty of Medicine, University of Oviedo, Oviedo, Asturias, Spain; 2 Department of Microbiology and Biochemistry of Dairy Products, Instituto de Productos Lácteos de Asturias (IPLA), Consejo Superior de Investigaciones Científicas (CSIC), Villaviciosa, Asturias, Spain

## Abstract

Intestinal dysbiosis, characterized by a reduced *Firmicutes*/*Bacteroidetes* ratio, has been reported in systemic lupus erythematosus (SLE) patients. In this study, *in vitro* cultures revealed that microbiota isolated from SLE patient stool samples (SLE-M) promoted lymphocyte activation and Th17 differentiation from naïve CD4^+^ lymphocytes to a greater extent than healthy control-microbiota. Enrichment of SLE-M with Treg-inducing bacteria showed that a mixture of two Clostridia strains significantly reduced the Th17/Th1 balance, whereas *Bifidobacterium bifidum* supplementation prevented CD4^+^ lymphocyte over-activation, thus supporting a possible therapeutic benefit of probiotics containing Treg-inducer strains in order to restore the Treg/Th17/Th1 imbalance present in SLE. In fact, *ex vivo* analyses of patient samples showed enlarged Th17 and Foxp3^+^ IL-17^+^ populations, suggesting a possible Treg-Th17 trans-differentiation. Moreover, analyses of fecal microbiota revealed a negative correlation between IL-17^+^ populations and *Firmicutes* in healthy controls, whereas in SLE this phylum correlated directly with serum levels of IFNγ, a Th1 cytokine slightly reduced in patients. Finally, the frequency of *Synergistetes*, positively correlated with the *Firmicutes*/*Bacteroidetes* ratio in healthy controls, tended to be reduced in patients when anti-dsDNA titers were increased and showed a strong negative correlation with IL-6 serum levels and correlated positively with protective natural IgM antibodies against phosphorylcholine.

Systemic lupus erythematosus (SLE) is a chronic autoimmune disease triggered by a combination of environmental and genetic factors that result in a breakdown in tolerance towards self-antigens^1^. The subsequent production of autoantibodies by autoreactive B cells constitutes a key pathological factor in SLE, since it leads to the formation and deposition of immune-complexes that cause tissue damage^2^. Likewise, naïve CD4^+^ cells activated by recognition of such self-antigens can be differentiated into several subsets based on the pattern of cytokines present in the local environment^3^. In addition to the well-known paradigm of Th1/Th2 cell immune response, nowadays much evidence reveals the presence of alterations in Th17 and regulatory T (Treg) cells in SLE disease^4^,^5^,^6^. With regard to Th17cells, some studies support their pivotal role as primary drivers of autoimmune responses in SLE through the secretion of proinflammatory cytokines involved in local inflammation and tissue destruction, including IL-17, IL-22 andIL-23^7^,^8^. Accordingly, increased circulating levels of IL-17 and IL-17-producing T cells have been recently reported in SLE^9^,^10^,^11^. Moreover, IL-17-producing T cells have also been shown to infiltrate the lungs, skin and kidneys in lupus patients, contributing to organ damage^10^,^12^. Conversely, Treg cells are essential for preventing autoimmune and inflammatory diseases, since they present a suppressive activity on aberrant effector responses^13^. Naturally occurring Treg cells emerge from the thymus and are primarily characterized by the presence of high levels of CD25 (IL2Rα chain) and FOXP3, a transcription factor required for the development and function of Treg cells^14^. In addition, Treg cells could be expanded or induced in peripheral tissues in response to diverse antigens^15^. Most studies report either reduced numbers or impaired function of circulating Treg cells in SLE patients^16^,^17^,^18^.

Increasing evidence suggests that the composition of the commensal microbiota colonizing the gut affects the differentiation of immune cells present in the gut-associated lymphoid tissues (GALTs)^19^. Specifically, plasmatic cells in the lamina propria are involved in the production of T cell-independent antibodies against components of both commensal and pathogenic bacteria as well as apoptotic cells, named natural IgM antibodies^20^. Interestingly, several studies have reported immunoregulatory functions of natural IgM antibodies inhibiting the inflammatory signaling in innate immune cells and suppressing autoimmune disease^21^,^22^. On the other hand, after the recognition of bacterial antigens, gut dendritic cells (DCs) may induce the differentiation of naïve CD4^+^ T cells into different types of effector or regulatory T cells^23^,^24^,^25^,^26^. Under physiologic conditions, the normal microbiota presented in healthy individuals favors the maintenance of the intestinal immune homeostasis^27^. Conversely, several studies suggest that alterations in the gut microbiota composition, known as dysbiosis, may be a critical factor in the development of numerous immune-mediated pathologies, probably in disease-susceptible hosts, through the generation of an imbalance between Th and Treg cells^19^,^28^,^29^,^30^,^31^. In this sense, intestinal dysbiosis has been associated with the development of several autoimmune diseases, including inflammatory bowel disease, type 1 diabetes, rheumatoid arthritis and multiple sclerosis^32^,^33^,^34^,^35^,^36^,^37^,^38^. In this regard, we have recently described a SLE-associated intestinal dysbiosis characterized by a significantly lower *Firmicutes* to *Bacteroidetes* ratio, the most abundant phyla in the human gut^39^ that has been previously described as imbalanced in other disorders^37^,^38^,^40^. Since these studies suggest that microbiota could control the Th/Treg axis outside the gut, the immune stimulation by specific bacteria could have a beneficial effect on inflammatory diseases^33^. Thus, it is known that some bacterial strains might induce the generation of Treg cells (iTreg) from naïve precursors^23^,^41^,^42^,^43^. Specifically, accumulating evidence supports the role of commensal strains of *Bifidobacterium* and *Clostridium* spp. belonging to clusters IV and XIVa in the induction of Treg cells^23^,^41^,^42^,^43^.

The study aims to evaluate the influence of fecal microbiota obtained from SLE patients and healthy controls in the *in vitro* differentiation of Th and Treg populations as well as the possible effect of enriching SLE gut microbiota with bifidobacteria and Clostridia strains known to be inducers of Treg cells. Then, we analyzed the possible relationship between the SLE-associated gut dysbiosis and the presence of immune parameters characteristic of these patients, such as the Treg/Th populations, cytokine levels, disease activity and the production of both pathogenic anti-dsDNA and protective natural IgM anti-phosphoryl choline antibodies.

## Results

### *In vitro* effect of SLE fecal microbiota on Treg/Th differentiation

Given the gut dysbiosis recently reported in SLE patients^39^, we aimed to evaluate the influence of fecal microbiota obtained from SLE patients (SLE-M) and healthy controls (HC-M) in the *in vitro* differentiation of regulatory T cells (Treg), as well as Th1 and Th17 effector populations from naïve CD4^+^ T lymphocytes. In addition, to estimate the effect of enriching the gut microbiota with strains able to increase the Treg subset, 5, 10 or 30% of SLE-M were replaced with the same amounts of *Bifidobacterium bifidum* LMG13195 (Bb), a strain known to induce Foxp3 expression^23^,^41^, or with a mixture of two Clostridia strains (Cl) with a putative Treg-inducer effect (*Ruminococcus obeum* DSM25238 and *Blautia coccoides* DSM935)^42^,^43^. Thus, immature monocyte-derived DCs were treated for 48 h with LPS, as a maturation control, or with the different bacterial preparations and then used to prime CD4^+^CD45RA^+^ naïve T lymphocytes. After 12 days of culture in the presence of IL-2, Treg (CD4^+^CD25^high^CD127^low^Foxp3^+^), Th17 and Th1(IFNγ- and IL-17-expresing cells, respectively) populations were determined by flow cytometry in CD4^+^ lymphocytes.

**Figure 1:**
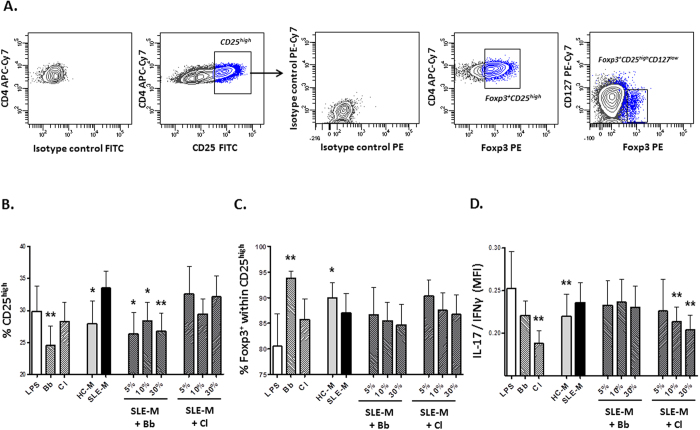
Fecal microbiota isolated from SLE patients influences Treg/Th differentiation. Naïve CD4^+^CD45RA^+^ lymphocytes were co-cultured for 12 days with DC previously maturated with LPS (maturation control), fecal microbiota isolated from healthy controls (HC-M) or SLE patients (SLE-M) as well as with *Bifidobacterium bifidum* LMG13195 (Bb), Clostridia strains (Cl) or SLE-M containing 5, 10 or 30% Bb or Cl. Cultured CD4^+^ T cells were recovered, stained for Treg markers, IL-17 and IFNγ and analyzed by flow cytometry. (**A**) Sequential gating strategy used to identify Treg cells (CD4^+^CD25^high^CD127^low^Foxp3^+^ ). Positive cells for each marker were determined using fluorescence of cells labelled with the corresponding isotype-matched conjugated irrelevant MAb as a negative control. CD4^+^ T cells showing the highest CD25 expression were identified as CD4^+^CD25^high^. Then, CD25^high^ population was analyzed to determine the proportion of cells expressing Foxp3 (Foxp3^+^ within CD25^high^) as well as the amount of Treg cells (CD25^high^CD127^low^Foxp3^+^). Density plots correspond to a representative experiment. Analyses of (**B**) CD25^high^population, (**C**) Foxp3^+^ cells within CD25^high^ population, and (**C**) IL-17/IFNγ expression. Bars represent the mean and SEM of seven independent experiments performed with different blood donors. Statistical differences between SLE-M and the different treatments were evaluated by the Wilcoxon test for paired data. *p < 0.1; **p < 0.05.

As expected, stimulation and expansion with IL-2 induced IL-2Rα (CD25) expression in most CD4^+^ lymphocytes (Fig. 1A), however the amount of cells presenting elevated CD25 levels (CD25^high^) showed differences among treatments (Fig. 1B). Specifically, SLE-M cultures tended to generate more CD25^high^ cells than HC-M, whereas the lowest levels of this population were obtained after stimulation with the Bb strain. In fact, the upregulatory effect of SLE-M on CD25 expression was reverted after Bb supplementation. Regarding Treg cells (CD4^+^CD25^high^ CD127^low^Foxp3^+^), no significant differences were observed between HC-M or SLE-M stimulation. However, the proportion of Foxp3^+^ cells included within the CD25^high^ population was lower in SLE- than in HC-M derived cultures (Fig. 1C), thus suggesting that part of the CD25^high^ cells generated in the presence of SLE-M were activated lymphocytes rather than Treg cells. Unexpectedly, although Bb increased Foxp3^+^ cells notably, supplementation of SLE-M with this strain did not increase the generation of Foxp3^+^ cells within the CD25^high^ subset.

Finally, although no significant differences were detected in IL-17 or IFNγ expression between cells treated with both fecal microbiotas, the IL-17/IFNγ ratio was significantly higher in SLE- than in HC-M cultures, whereas the lowest ratio was induced by Cl-conditioned DCs. Moreover, SLE-M supplementation with Cl, but not with Bb, induced a significant dose dependent reduction of the IL-17/IFNγ balance (Fig. 1D). Therefore, *in vitro* stimulation of naïve CD4^+^ T cells with SLE-M seems to promote lymphocyte activation and Th17 differentiation to a greater extent than HC-M treatment, thus supporting Th17/Treg disturbances in SLE. In addition, enrichment of SLE microbiota with Bb may prevent lymphocyte activation whereas Cl supplementation restores Th17/Th1 balance.

### Relationship between immune parameters and fecal microbiota in SLE patients

To know whether the Treg/Th responses elicited *in vitro* by SLE-M could reflect the characteristic immune features of SLE patients, we analyzed Foxp3, CD25, CD127, IL-17 and IFNγ expression in fresh peripheral blood CD4^+^ lymphocytes as well as the serum levels of a battery of cytokines in 37 SLE patients and 36 HC (Table 1). In addition, since 40 of these individuals (20 SLE and 20 HC) have been previously included in a metagenomic study of fecal microbiota^39^ (Table 2), we determined the possible relationship between these immune parameters and the SLE-associated gut dysbiosis.

**Figure 2:**
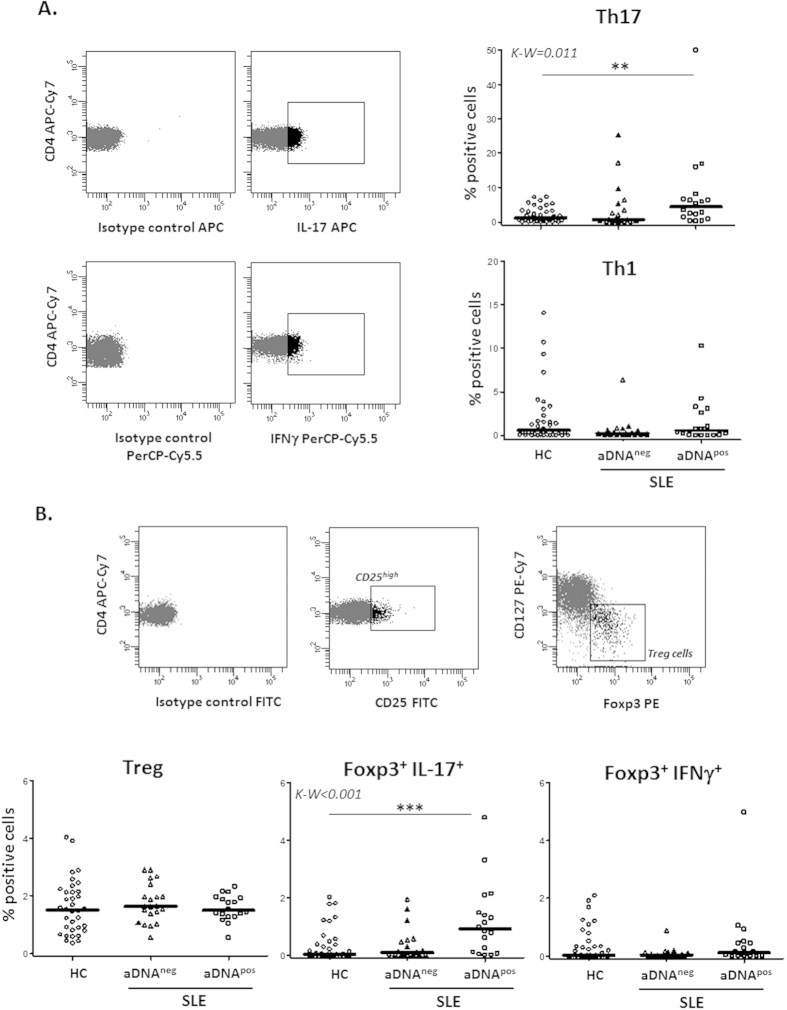
Increased IL-17 producing cells in SLE patients with anti-dsDNA antibodies. Foxp3, CD25, CD127, IL-17 and IFNγ expression was analyzed in fresh peripheral blood CD4^+^ lymphocytes from SLE patients and HC. (**A**) Dot-plots show cells positive for IL-17 or IFNγ expression, determined attending to the fluorescence of cells labelled with the corresponding isotype-matched conjugated irrelevant MAb as a negative control. Scatter plots represent the percentage of IL-17^+^ (Th17) and IFNγ^+^ (Th1) CD4^+^ cells in HC and SLE patients presenting (*pos*) or not (*neg*) anti-dsDNA antibodies (aDNA). Horizontal bars show the median. (**B**) Treg cells were sequentially identified as CD4^+^CD25^high^CD127^low^Foxp3^+^ cells. Scatter plots represent the quantity of Treg, Foxp3^+^ IL-17^+^ and Foxp3^+^ IFNγ^+^ cells in HC and SLE patients in function of their anti-dsDNA status, and horizontal bars show the median. Statistical differences among groups were evaluated by Kruskal-Wallis test and Dunn’s post test was conducted to determine which groups’ pairs had different means. **p < 0.01; ***p < 0.001.

Results showed an increased frequency of Th17 cells in SLE patients compared to HC, especially in those presenting anti-dsDNA antibodies, whereas no significant differences were observed in the Th1 subset (Fig. 2A). Moreover, Foxp3^+^ IL-17^+^ double positive cells were significantly increased in SLE patients compared with HC, the highest levels of such cells being observed again in those patients presenting anti-dsDNA antibodies (Fig. 2B). No significant differences were detected in the percentage of Treg cells (CD4^+^ CD25^high^ CD127^low^Foxp3^+^), suggesting that a Treg-Th17 trans-differentiation process could be involved in the development of Foxp3^+^ cells without regulatory activity in SLE patients.

On the other hand, the analysis of fecal microbiota in the HC group revealed a negative correlation between the frequency of *Firmicutes* and the size of the Th17 subset, most notably in Foxp3^+^ IL-17^+^ cells, whereas the opposite occurred with *Bacteroidetes* (Table 3); all these correlations were confirmed by multivariate linear regression analyses adjusted by weight, BMI and blood lipids (*p < 0.05, R^2^ > 0.6). However, such associations were completely absent in patients, in agreement with our previously described reduction of the *Firmicutes/Bacteroidetes* ratio in SLE.

**Figure 3:**
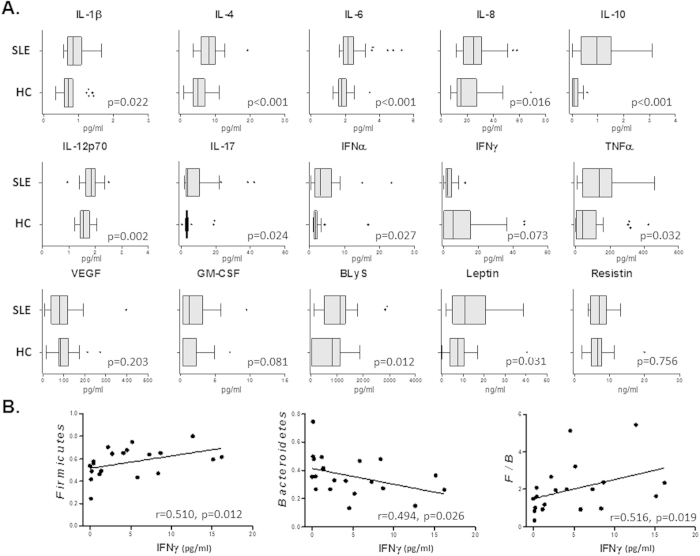
IFNγ serum levels in SLE patients were associated with *Firmicutes* to *Bacteroidetes* ratio. (**A**) Box and whiskers represent median and interquartile range of circulating amounts of cytokines in SLE patients compared to controls. Differences between both groups were assessed by the non-parametric Mann-Whitney *U* test. (**B**) Correlations between serum levels of IFNγ and the frequency of *Firmicutes*, *Bacteroidetes* or the *Firmicutes* to *Bacteroidetes* ratio (F/B) in SLE patients were evaluated using Spearman test.

Regarding cytokine serum levels, SLE patients presented higher amounts of IL-1β, IL-4, IL-6, IL-8, IL-10, IL-12p70, IL-17A, IFNα, TNFα, GM-CSF, BLyS and leptin than HC, whereas IFNγ showed a clear tendency to reduction (Fig. 3A). Interestingly, none of the increased or unchanged cytokines in SLE displayed significant associations with *Firmicutes* or *Bacteroidetes*, however, IFNγ levels correlated negatively with *Bacteroidetes* and positively with *Firmicutes* and the *Firmicutes/Bacteroidetes* ratio in patients (Fig. 3B). These associations were not detected in HC.

### Association between the frequency of *Synergistetes* and the presence of natural antibodies

As we previously reported^39^, the proportion of fecal *Synergistetes* did not show significant differences between SLE patients and healthy controls. However, we found a negative correlation trend between this bacterial group and the titer of anti-dsDNA antibodies (r = −0.386, p = 0.084), not detected with any other of the previously analyzed microbial groups. Likewise, serum levels of IL-6 associated negatively with the amount of *Synergistetes* in SLE patients (r = −0.738, p < 0.001), thus suggesting a possible relationship between this bacterial group and disease activity or antibody production. Therefore, aiming to expand the knowledge about the possible role played by intestinal *Synergistetes* in the development of humoral immune responses, we quantified serum levels of anti-PC IgM, natural protective antibodies, and anti-PC IgG (lacking this effect) as well as total circulating IgM and IgG. No significant differences were detected in anti-PC antibodies (IgM and IgG) and total IgM between patients and controls, but the amount of total IgG was increased in patients compared to controls (p = 0.027). Interestingly, anti-dsDNA titer correlated negatively with both isotype anti-PC antibodies (IgM: r = −0.508, p = 0.019; IgG: r = −0.460, p = 0.036) and with total IgM (r = −0.501, p = 0.021) but not IgG (r = 0.065, p = 0.780) levels, thus suggesting a detrimental effect of SLE disease activity on naturally occurring protective IgM antibodies.

On the other hand, *Synergistetes* exhibited a positive correlation with total and anti-PC IgM in SLE patients and with total and anti-PC IgM/IgG ratio in patients and controls (Table 4). Interestingly, serum levels of IL-6 in patients showed the opposite associations. All these results suggest that intestinal *Synergistetes* may promote the development of protective natural IgM antibodies, an effect particularly relevant in SLE patients since the increased levels of anti-dsDNA and/or IL-6 could downregulate this bacterial group.

Finally, it is noteworthy that *Synergistetes* correlated negatively with *Bacteroidetes* (r = −0.486, p = 0.022) and positively with the *Firmicutes/Bacteroidetes* ratio (r = 0.443, p = 0.039) in controls, but not in SLE patients (r = 0.075, p = 0.745 and r = −0.136, p = 0.556, respectively), supporting the role of these bacterial groups in SLE.

## Discussion

Intestinal dysbiosis has been associated with several immune mediated diseases, including SLE^39^ and other autoimmune conditions^32^,^33^,^34^,^35^,^36^,^37^,^38^, pointing out a possible role of the microbiome in the balance between inflammatory and regulatory responses, either in the intestine or systemically. Therefore, understanding how the gut microbiota shapes immune responses seems to be critical for human health, particularly in chronic inflammatory disorders such as autoimmune diseases. However, to the best of our knowledge, this is the first study evaluating the effect of intestinal microbiota isolated from SLE stool samples on the induction of effector or regulatory T cell responses.

Our findings revealed that the differences in the composition of the fecal microbiota between SLE patients and HC elicited different *in vitro* immune responses. Specifically, DCs stimulated with SLE-M promoted Th17 differentiation from naïve CD4^+^ T lymphocytes to a greater extent than HC-M conditioned DCs. No differences were detected in the generation of Treg cells, but the enlarged CD25^high^ population obtained with SLE-M treatment tended to include a lower proportion of Foxp3^+^ cells, supporting their activated rather than regulatory status. Hence, the altered SLE gut microbiota could enhance lymphocyte activation and Th17 differentiation, thus sustaining the preexisting inflammation.

These findings are in accordance with the results of the *ex vivo* analyses, since the frequency of Th17 cells was increased in fresh peripheral blood from SLE patients compared to HC, especially in those presenting anti-dsDNA antibodies, whereas no differences were observed in Treg cells. Interestingly, SLE patients exhibited a higher proportion of Foxp3^+^ cells producers of IL-17, thus suggesting a possible Treg-Th17 trans-differentiation process that could be responsible for the reduced regulatory activity of Treg cells reported in SLE patients^18^. Similarly, an increased prevalence of circulating IL-17 and Foxp3 double-expressing CD4^+^ lymphocytes, together with a reduced Treg suppressor ability, have been found in patients with inflammatory bowel disease^44^. The elevated frequency of Treg cells present in the gut compared with other organs^45^ is noteworthy, but they have an increased Treg/Th plasticity which could be influenced by inflammatory mediators, specific bacterial strains and other micro-environmental factors^46^. In line with this, the analyses of fecal microbiota revealed a strong negative correlation between the size of IL-17^+^ Foxp3^+^, and to a lesser extend Th17, populations and the frequency of *Firmicutes* in healthy controls, suggesting that they could counteract Th17 differentiation. On the other hand, serum levels of IFNγ, the prototypic Th1 cytokine and slightly reduced in SLE, correlated directly in patients with the amount of *Firmicutes* and with the *Firmicutes* to *Bacteroidetes* ratio, this imbalance being the main feature of SLE dysbiosis and independent to disease duration, lifestyle and dietary-related factors^39^. Therefore, we hypothesize that bacterial strains belonging to the *Firmicutes* phylum could be involved in the generation and/or maintenance of functional Treg cells in the gut, avoiding the trans-differentiation into effector Th17 cells in physiological conditions, whereas a diminished proportion of these bacteria in pathological situations may promote Th17 vs Th1 and Treg bias and generate IL-17^+^ Foxp3^+^ cells. Thus, enrichment of gut microbiota in Treg-inducing bacteria could be a desirable goal for SLE patients. However, although *Firmicutes/Bacteroidetes* ratio represents the main difference between healthy controls and SLE patients, the specific bacterial groups implicated in the observed Th17 immune response associated to SLE microbiota could not be determined by our approach, even more having into account that *Firmicutes* phyla includes both Th17^47^ and Treg^23^,^41^,^42^,^43^,^44^ inducing bacteria. Hence, the confirmation of such hypothesis would benefit from additional experiments using germ-free mice or fecal microbiota transplant procedures in animal models.

Certain commensal microorganisms have demonstrated a Treg-inducer capability, and therefore have been proposed as potential probiotic strains appropriate for the modulation of an excessive inflammatory response to restore the immune homeostasis at the intestinal mucosa. Hence, in this study we performed *in vitro* analyses aiming to determine the possible effect of enriching SLE gut microbiota with strains known to be able to differentiate naïve T lymphocytes into Treg cells. *Firmicutes* phylum contains several *Clostridium* spp., belonging to clusters IV and XIVa, known to induce Treg cells^23^,^41^,^42^,^43^. Similarly, bifidobacteria have been also demonstrated to promote the Treg polarization and Th17 reduction *in vivo* using murine models of colitis^48^. Therefore, we used a mixture of two of these Clostridia strains, as well as a *Bifidobacterium* strain with previously demonstrated *in vitro* ability to generate functional Treg cells^23^,^24^. Unexpectedly, neither was able to increase this population, but results showed other beneficial effects, different for each bacterial treatment. Supplementation of SLE-M with Clostridia induced a significant dose-dependent reduction of the IL-17/IFNγ balance, thus restoring Th1 bias, whereas bifidobacteria enrichment prevented the over-activation of CD4^+^ lymphocytes, as detected by the reduction of CD25 expression. These effects, however, could be the consequence of an active suppression of effector Th cells. In fact, the observed down regulation of Th17 cells may be explained by a preferential promotion of Treg cells by Clostridia strains, in accordance with the results obtained by Atarashi *et al.*^43^ using germ-free mice colonized with different fractions of healthy human microbiota. Likewise, suppressive function of Treg cells generated with the *Bifidobacterium* strain used here results in downregulation of CD25 expression on effector cells^23^. Therefore, in view of these results and the previously reported SLE dysbiosis, it seems reasonable to consider the possible therapeutic benefit of the supplementation with probiotics containing Treg-inducer strains in order to restore the Treg/Th17/Th1 balance in SLE patients.

Another remarkable result is the suggested role played by *Synergistetes*, a little known intestinal bacterium, in SLE. This bacterial group correlated negatively with *Bacteroidetes* and positively with the *Firmicutes* to *Bacteroidetes* ratio in healthy controls, whereas in SLE patients a strong negative correlation was shown with serum levels of IL-6, a proinflammatory and Th17 promoting cytokine increased in SLE patients. Moreover, the amount of *Synergistetes* tended to be reduced when the titer of anti-dsDNA antibodies were increased, characteristic of SLE. These findings led us to evaluate the possible role of intestinal *Synegistetes* in the development of protective humoral immune responses. Natural IgM antibodies against commensal bacteria or neoantigens from apoptotic cells are commonly present in the human circulation from birth and have protective and immunoregulatory functions. Among them, IgM antibodies that recognize phosphorylcholine (PC) are valuable components of the immune system known to increase the phagocytosis of apoptotic cells and inhibit inflammatory pathways in autoimmunity and atherosclerosis^20^,^22^,^49^. However, anti-PC IgG did not seem to possess these protective effects. Results in our SLE cohort revealed that the proportion of *Synergistetes* correlated positively with total and anti-PC IgM and IgM/IgG ratio in SLE patients, whereas serum levels of IL-6 exhibited the opposite associations. These data suggest a protective role of intestinal *Synergistetes* promoting the generation of natural IgM antibodies, which could be hampered in SLE patients, especially those with high IL-6 levels. Interestingly, it has been reported that anti-PC IgM antibodies can counteract IL-6 upregulation *in vitro* and *in vivo*^22^, probably by the inhibition of MAPK responses to TLR agonists, including lupus immune complexes^50^, thus explaining the opposite associations of these natural antibodies with *Synergistetes* and IL-6. Moreover, B1 cells and anti-PC antibodies are increased in IL-6 knockout mice whereas the opposite occurs with B2 cells and IgG levels^51^. In line with these results, the anti-dsDNA titer correlated negatively with anti-PC levels, thus supporting a deleterious effect of disease activity on the amount of natural protective antibodies. In fact, higher anti-PC IgM levels have been associated with lower SLE disease activity^20^. Unfortunately, active patients were not included in our study of the intestinal microbiota to avoid interference with the treatments, so no significant associations were detected between SLEDAI score and *Synergistetes* or anti-PC IgM antibodies.

The involvement of *Synergistetes* in the promotion of natural antibodies, and especially those with an atheroprotective role, could be of clinical relevance for patients with SLE and other autoimmune diseases, since they usually develop premature atherosclerosis and have an increased cardiovascular risk not explained by classical factors. It has been reported that diminished levels of anti-PC IgM antibodies in SLE patients could predict a subclinical cardiovascular disease^21^. Moreover, patients that have suffered a cardiovascular event presented significantly lower levels of these antibodies compared to the rest of patients^21^,^49^. In fact, therapy based on passive transfer of anti-PC has been used to inhibit atherosclerosis development^52^. Therefore, the identification of intestinal microorganisms able to expand natural humoral responses through the promotion of protective IgM antibodies, as may be *Synergistetes*, could be valuable tools to design clinical interventions in order to improve both disease activity and prevention of cardiovascular complications. In this sense, the analysis of fecal *Synergistetes* in SLE patients with and without cardiovascular events should be of interest.

In summary, our *in vitro* cultures with fecal microbiota isolated from SLE patients suggested that immune responses against intestinal bacteria could be involved in the lymphocyte over-activation as well as in the Treg-Th17 trans-differentiation observed in SLE patients, with the reduced *Firmicutes* to *Bacteroidetes* ratio probably having a role in this process. The altered immune responses associated with the intestinal dysbiosis could be reestablished, at least in part, by the supplementation with beneficial bacterial strains able to induce suppressor responses. In addition, our results revealed a possible role of intestinal *Synergistetes* in the development of natural protective anti-PC IgM antibodies. This could be of special relevance in patients with high IL-6 and/or anti-dsDNA levels, since they present low frequency of these bacteria.

## Methods

### Ethics approval

Ethics approval for this study was obtained from the Bioethics Committee of CSIC (Consejo Superior de Investigaciones Científicas) and from the Regional Ethics Committee for Clinical Research (Servicio de Salud del Principado de Asturias), according to the Declaration of Helsinki. All methods were carried out in accordance with the approved guidelines and signed informed written consent was collected from all participants prior to participation in the study.

### Generation of monocyte-derived DCs

Human peripheral blood mononuclear cells (PBMCs) were obtained from standard buffy-coat preparations from 7 healthy blood donors (Asturian Blood Transfusion Center, Oviedo, Spain) by centrifugation over Ficoll-Hypaque gradients (Lymphoprep, Nycomed, Oslo, Norway). Monocytes (CD14^+^ ≥ 95%) were isolated from previously obtained PBMCs by negative selection using the Human Monocyte enrichment kit (EasySep, Stem Cell Technologies, Canada).

Immature DCs were obtained from isolated monocytes by standard procedures. Thus, monocytes were cultured in 24-wellplates at 5 × 10^5^ cells/ml for 7 days at 37 °C and 5% CO_2_ in complete RPMI medium (RPMIc) [RPMI 1640 containing 2 mML-glutamine and 25 mM Hepes (Bio Whitaker, Verviers, Belgium), supplemented with 10% heat-inactivated fetal calf serum (FCS) and the antibiotics streptomycin and ampicillin at 100 mg/ml] in the presence of recombinant human (rh) IL-4 (35 ng/ml) and rhGM-CSF (70 ng/ml) (R&D Systems, Abingdon, UK). At days 2 and 5, 0.5 ml of the medium was removed without disturbing the clusters of developing DC and 0.5 ml of freshly made GM-CSF- and IL-4-containing medium was added to the wells, restoring the final volume in each well to 1 ml. At day 7, immature DCs were recovered, washed and resuspended in RPMIc medium at 5 × 10^5^ cells/ml for subsequent maturation.

### Stimulation of monocyte-derived DCs with isolated fecal microbiota

Immature monocyte-derived DCs were cultured in RPMIc medium with 1 mg/ml LPS from *E. coli* 0111:B4 (Sigma, St. Louis, MO), as a positive control of maturation, or with different bacterial preparations at a DC:bacteria ratio of 1:10. To this end, a pooled fecal microbiota (M) isolated from either four healthy controls (HC-M) or five SLE patients (SLE-M), previously shown to be representative of both conditions by metagenomics studies^39^, were prepared and used in DC cultures. Also, SLE-M were enriched with different proportions of *Bifidobacterium bifidum* LMG13195 (Bb), a strain known to induce Foxp3 expression^23^,^41^, or with a mixture of two Clostridia strains (Cl: *Ruminococcus obeum* DSM25238 and *Blautia coccoides* DSM935, ratio 1:1), which have a putative Treg-inducer effect^42^,^43^. Thus, 5, 10 or 15% SLE-M were replaced with the same proportions of Bb or Cl and used for DCs stimulation. Cultures with Bb and Cl bacterial preparations were performed as controls. After 48 hours, DCs from the 11 different treatments were harvested, the mature phenotype tested and used to stimulate naïve CD4^+^ T cells.

### Naïve CD4^+^ T cell stimulation with microbial conditioned-DCs

CD4^+^ T cells were isolated from PBMCs by negative selection using the Human CD4^+^ T Cell Enrichment Kit (EasySep), following the manufacturer’s instructions, and then naïve CD45RA^+^ T cells were separated after depletion of CD45RO^+^ cells (Miltenyi Biotec, Germany). Monocyte-derived DCs maturated with LPS or with the different bacterial preparations were cocultured with purified CD4^+^ CD45RA^+^ T cells in 48-well plates at DC:T cell ratio of 1:10. At day 5 and 8, cells incubated with all treatments were expanded with IL-2 (30 U). After 12 days, cells were collected and washed twice before analysis of the Treg/Th17/Th1 phenotype by flow cytometry.

### Bacterial strains and growth conditions

*Bifidobacterium bifidum* LMG13195 was grown in de MRS broth (Difco, Detroit, MI) supplemented with 0.05% (w/v) L-cysteine (Sigma). *Ruminococcus obeum* DSM25238 and *Blautia coccoides* DSM935 were cultivated in a combination of Reinforced Clostridial Broth (Merck, Darmstadt, Germany) and Brain-Heart Infusion (Difco), supplemented with 5% (v/v) heat-inactivated FCS (LabClinics, Barcelona, Spain). Cultures were grown at 37 °C in a MG500 anaerobic chamber (Don Whitley Scientific, West Yorkshire, UK) with an atmosphere of 10% (v/v) H_2_, 10% CO_2_, and 80% N_2_. Cultures were harvested by centrifugation, washed three times in phosphate buffered saline (PBS) and resuspended in the same buffer to a concentration of 10^8^ bacteria/ml. Bacterial were counted by using a Thoma cell counting chamber (Marienfeld Superior, Germany). Bacterial cells were killed by exposing them to three consecutive cycles of 30 minutes under radiation in a UV chamber (15 W, Selecta, Barcelona, Spain). Plate counting was carried out after UV treatment to corroborate the absence of bacteria able to recover in the proper medium. UV-killed bacterial suspensions were distributed in aliquots, and stored at −80 °C until use. The identity of the strains was confirmed by sequencing the V1 and V2 variable regions of the 16 S rRNA gene using primers plb16 (5′-AGAGTTTGATCCTGGCTCAG-3′) and mlb16 (5′-GGCTGCTGGCACGTAGTTAG-3′)^53^.

### Stool samples and microbiota separation

One portion of stool sample of 4 healthy controls and 5 SLE patients, previously used in a metagenomic study^39^, was submitted to a density gradient centrifugation to separate the microbiota from the rest of the fecal material, according to the method of Courtois *et al.*^54^. Feces were homogenized in sterile NaCl 0.9% (1:9; w/v) for 1 min in a homogenizer (Stomacher Lab Blender 400, WVR, Barcelona, Spain). A solution of Nycodenz^®^ 80% (w/v) (PROGEN Biotechnik GmbH, Heidelberg, DE) was prepared in ultrapure water, and sterilized at 121 °C for 15 min. A volume of 10.5 ml of the homogenized fecal samples was placed on the top of 3.5 ml of the Nycodenz^®^ solution, and centrifuged at 10,000 g for 40 min at 4 °C. The upper phase, containing soluble debris, was discarded and the layers corresponding to the microbiota were kept in ice for 5 min, in order to allow non-soluble debris to precipitate, washed twice in PBS, and stored in the same buffer in aliquots of 1 ml of 10^8^ microorganisms/ml at −80 °C. UV-inactivated microbiota was obtained as described in the previous section.

### SLE patients and healthy controls

Thirty-seven SLE patients fulfilling at least four American College of Rheumatology (ACR) revised criteria for the classification of SLE^55^ were selected from the updated Asturian Register of Lupus^56^,^57^. Information on clinical features during the disease course was obtained by reviewing clinical histories (Table 1). Thirty-six sex and age-matched healthy blood donors were used as controls (mean age ± SD: 42.56 years ± 11.39). At the time of sampling, anti-dsDNA titer, SLE disease activity index (SLEDAI) and/or weight, BMI (body mass index) and blood lipids [Triglycerides, HDL (high-density lipoprotein), LDL (low-density lipoproteins) and total cholesterol] were evaluated and patients were asked precise questions regarding the treatment received over the previous 6 months.

### Intestinal microbiota analysis

Metagenomic analysis of fecal microbiota were performed in 20 non active SLE patients without antibiotic or immunosuppressive treatment in the last 6 months and 20 age-matched healthy controls as previously described^39^. Faecal DNA extraction, 16 S rRNA amplification sequencing of 16 S rRNA gene-based amplicons and the sequence-based microbiota analysis were reported elsewhere^39^. The raw sequences reported in this article are deposited in the NCBI Short Read Archive (SRA) (study accession number: SRP028162).

### Flow cytometric analysis

Phenotypic studies of cultured cells and blood samples were performed after staining with the appropriate monoclonalantibody (mAb). Maturation of cultured DCs was verified after staining for 30 min at 4 °C with anti -CD86 fluorescein isothiocyanate (FITC), -CD80 phycoerythrin (PE), -HLA-DR PE-Cy5, -CD1a FITC mAb, or with the corresponding isotype matched conjugated irrelevant mAb as a negative control (all mAb were supplied by Pharmingen). To determine Treg/Th17/Th1 phenotype both in cultured cells and blood samples [previously lysed with 2 ml BD Lysing Solution (BD Biosciences, San Diego, CA) for 5 minutes and washed twice with PBS], CD4^+^ lymphocytes were first extracellularly stained with anti-CD4 allophycocyanin-Cy7 (APC-Cy7), anti-CD25 FITC and anti-CD127 PE-Cy7mAb or with the corresponding isotype-matched conjugated irrelevant mAb (all from eBiosciences, San Diego, CA). Then, cells were fixed, permeabilized and intracellularly stained with anti-FOXP3 PE, IFNγ PerCP-Cy5.5 and IL-17 A APC (Foxp3/transcription factor staining buffer set; eBiosciences) or with the corresponding isotype-matched conjugated irrelevant mAb, following the manufacturer’s instructions. A minimum of 10,000 CD4^+^ lymphocytes were acquired on a FACSCanto II flow cytometer (BD) and analyzed using the FlowJo software (Tree Star Inc). The specific fluorescence intensity was quantified as the mean fluorescence intensity (MFI) calculated by subtracting the background of isotype matched control staining from the total fluorescence.

### Cytokine determination

Serum samples from SLE patients and HC were collected and maintained at −80 °C until cytokine determination was carried out. IL-1β, IL-4, IL-6, IL-8, IL-10, IL-12p70, IL-17 A, IFNα, VEGF and GM-CSF amounts were analyzed by Cytometric Bead Arrays Flex Set using a FACS Canto II flow cytometer (BD Biosciences). For IL-1β, IL-6, IL-10 and IL-12p70, an Enhanced Sensitivity Flex Set was needed. ELISA kits were used for the quantification of TNFα, leptin, resistin (Mini EDK kit, PeproTech), BLyS (Human BAFF Instant ELISA, eBioscience) and IFNγ (OptEIA kit, BD) following the manufacturer’s instructions. The lower limits of detection were 48.4 fg/ml for IL-1β, 1.4 pg/ml for IL-4, 68.4 fg/ml for IL-6, 1.2 pg/ml for IL-8, 13.7 fg/ml for IL-10, 12.6 fg/ml for IL- 12p70, 0.3 pg/ml for IL-17 A, 1.25 pg/ml for IFNα, 4.5 pg/ml for VEGF, 0.2 pg/ml for GM-CSF, 3.9 pg/ml for TNFα, 63 pg/ml for leptin, 24 pg/ml for resistin, 130 pg/ml for BLyS and 0.58 pg/ml for IFNγ.

### Anti-Phosphorylcholine antibodies quantification

IgG and IgM antibodies against Phosphorylcholine (anti-PC) were quantified in serum samples from patients and controls by an in-house ELISA test, as follows. Microtiter wells (Maxisorp, Nunc) were coated overnight with phosphorylcholine conjugated to bovine serum albumin (PC-BSA) (Biosearch Technologies, Petaluma) and blocked with PBS 2% BSA for 2 hours at 37 °C. A sera pool of healthy controls was used as anti-PC Ab standard. Serum samples and anti-PC standard were diluted in Tris Buffered Saline (TBS) and incubated for 2 hours at room temperature (RT). After washing with TBS/Tween 20 (0.05%), wells were incubated for 2 hours at RT with alkaline phosphatase-conjugated anti-human IgG or IgM (Immunostep, Salamanca, Spain). Finally, plates were washed twice and revealed using p-nitrophenylphosphate as substrate. Absorbance was determined at a wavelength of 405 nm. Quantities of serum anti-PC arbitrary units were calculated for each sample according to the standard curves. Similarly, total IgG or IgM were quantified by conventional ELISA techniques.

### Statistical analysis

The Kolmogorov-Smirnov test was used to assess the normal distribution of the data. *In vitro* experiments data were represented by mean ± SEM and differences between culture conditions were assessed by the paired Wilcoxon test. SLE patients and healthy controls cytokine serum levels were expressed as the median value (interquartile range) and non-parametric Mann-Whitney U-test was used to determine differences between both groups. Percentages of Treg/Th1/Th17 cells were compared by using the Kruskal-Wallis test; when a significant test was obtained, Dunn’s post hoc tests were conducted to determine statistical differences in pairs of groups. Associations of fecal microbial group’s frequencies with T cell subsets, cytokine serum levels and autoantibodies were examined by Spearman’s rank correlation test and confirmed by multivariate linear regression analyses adjusted by weight, BMI and blood lipids (triglycerides, HDL, LDL and total cholesterol]. GraphPad Prism 5 software (GraphPad Software, USA) and SPSS 22 statistical software package (SPSS Inc.) were used for all determinations, and a *p*-value < 0.05 was considered significant.

## Additional Information

**How to cite this article**: Lopez, P. *et al.* Th17 responses and natural IgM antibodies are related to gut microbiota composition in systemic lupus erythematosus patients. *Sci. Rep.* **6**, 24072; doi: 10.1038/srep24072 (2016).

## Figures and Tables

**Table 1:** Demographic and clinical features of SLE patients.

	SLE patients (N = 37)
Sex (female/male) (n)	36/1
Age, years (mean ± SD)	48.40 ± 12.99
Age at diagnosis, years (mean ± SD)	38.32 ± 13.64
Disease duration, years (mean ± SD)	8.48 ± 6.65
SLEDAI score (mean ± SD)	4.45 ± 3.28
Clinical manifestations, n (%)	
Malar rash	17 (45.95)
Discoid lesions	8 (21.62)
Photosensitivity	21 (56.76)
Oral ulcers	16 (43.24)
Arthritis	22 (59.46)
Serositis	6 (16.22)
Cytopenia	24 (64.86)
Renal disorder	11 (29.73)
Neurological disorder	2 (5.40)
Autoantibodies, n (%)	
ANAs	37 (100.00)
Anti-dsDNA/titer, U/ml (mean ± SD)	18 (48.65)/41.50 ± 61.31
Anti-SSa	19 (51.35)
Anti-SSb	3 (9.68)
Anti-Sm	4 (10.81)
Anti-RNP	4 (10.81)
Treatment, n (%)	
None or NSAIDs	4 (10.81)
Antimalarial drugs	30 (81.08)
Glucocorticoids	10 (27.03)
Immunosuppressive drugs^a^	3 (8.11)

dsDNA: double stranded DNA; NSAID: non-steroidal anti-inflammatory drug.

^a^Methotrexate and/or mycophenolate mophetil.

**Table 2:** SLE patients and healthy controls included in the intestinal microbiota analysis.

	SLE patients (N = 20)	Healthy controls (N = 20)
Sex (female/male) (n)	20/0	20/0
Age (years)	49.21 ± 10.70	46.92 ± 8.63
BMI (Kg/m^2^)	26.17 ± 5.34	25.20 ± 4.20
Weigth (Kg)	64.73 ± 11.23	67.28 ± 14.03
Blood lipids (mg/dL)		
Total cholesterol	195.85 ± 37.22	198.32 ± 34.34
HDL cholesterol	64.80 ± 15.52	63.00 ± 9.09
LDL cholesterol	116.35 ± 38.52	120,32 ± 31.38
Triglycerides	71.55 ± 31.02	73.73 ± 37.33
Disease parameters		
Age at diagnosis (years)	38.24 ± 11.85	
Disease duration (years)	10.40 ± 7.31	
SLEDAI score	4.83 ± 2.89	
Anti-dsDNA titer (U/ml)	22.01 ± 30.37	
Treatment, n		
None or NSAIDs	2	
Antimalarial drugs	18	

Values represent means ± SD. BMI: body mass index; HDL: high-density lipoprotein; LDL: low-density lipoproteins; dsDNA: double stranded DNA; NSAID: non-steroidal anti-inflammatory drug.

**Table 3:** Associations of *Firmicutes*, *Bacteroidetes* and the *Firmicutes* to *Bacteroidetes* ratio with different CD4^+^ T cell subsets in healthy controls.

CD4^+^ T subsets	*Firmicutes*	*Bacteroidetes*	*Firmicutes/Bacteroidetes*
Th1	r = 0.040	r = 0.183	r = −0.119
	p = 0.862	p = 0.427	p = 0.606
Th17	r = −0.444*	r = 0.294	r = −0.329
	**p = 0.044**	p = 0.196	p = 0.145
Foxp3^+^ IL-17^+^	r = −0.561*	r = 0.465*	r = −0.487*
	**p = 0.008**	**p = 0.034**	**p = 0.025**
Foxp3^+^ IFNγ^+^	r = 0.054	r = 0.156	r = −0.089
	p = 0.816	p = 0.500	p = 0.702

Correlation analyses were evaluated by Spearman test and confirmed by multivariate linear regression analyses adjusted by weight, BMI and blood lipids (*p < 0.05, R^2^ > 0.6).

**Table 4:** Relationship of fecal *Synergistetes* and IL-6 serum levels with anti-PC and total IgM and IgG antibodies.

	*Synergistetes*		IL-6	
	HC	SLE	HC	SLE
**IgM:**				
anti-PC	r = 0.404	r = 0.624*	r = −0.004	**r =** −**0.473**
	p = 0.062	**p = 0.002**	p = 0.986	**p = 0.030**
total	r = 0.101	r = 0.824*	r = 0.081	r = −0.642*
	p = 0.662	**p < 0.001**	p = 0.729	**p = 0.002**
**IgG:**				
anti-PC	r = −0.156	r = 0.239	r = −0.049	r = −0.038
	p = 0.523	p = 0.286	p = 0.842	p = 0.286
** total**	r = −0.301	r = −0.078	r = 0.133	r = 0.332
	p = 0.211	p = 0.737	p = 0.586	p = 0.142
**IgM/IgG ratio:**				
** anti-PC**	**r = 0.670**	r = 0.495*	r = −0.005	**r = −0.488**
	**p = 0.002**	**p = 0.023**	p = 0.983	**p = 0.025**
** total**	**r = 0.496**	r = 0.597*	r = −0.082	r = −0.610*
	**p = 0.031**	**p = 0.004**	p = 0.737	**p = 0.003**

Correlation analyses were evaluated by Spearman test and confirmed by multivariate linear regression analyses adjusted by weight, BMI and blood lipids (*p < 0.05, R^2^ > 0.6).
